# Seasonality, Interannual Variability, and Linear Tendency of Wind Speeds in the Northeast Brazil from 1986 to 2011

**DOI:** 10.1155/2013/490857

**Published:** 2013-10-22

**Authors:** Alexandre Torres Silva dos Santos, Cláudio Moisés Santos e Silva

**Affiliations:** ^1^Program Post-Graduate in Climate Sciences, Center of Exact Sciences, Federal University of Rio Grande do Norte, Campus Universitário, s/n, 59072-970 Natal, RN, Brazil; ^2^Center for Gas Technology and Renewable Energy (CTGAS-ER), Avenida Capitão-Mor Gouveia, 1480 Lagoa Nova, 59063-400 Natal, RN, Brazil; ^3^Department of Theoretical and Experimental Physics (DFTE), Federal University of Rio Grande do Norte (UFRN), Campus Universitário, s/n, 59072-970 Natal, RN, Brazil

## Abstract

Wind speed analyses are currently being employed in several fields, especially in wind power generation. In this study, we used wind speed data from records of Universal Fuess anemographs at an altitude of 10 m from 47 weather stations of the National Institute of Meteorology (*Instituto Nacional de Meteorologia*-INMET) from January 1986 to December 2011. The objective of the study was to investigate climatological aspects and wind speed trends. To this end, the following methods were used: filling of missing data, descriptive statistical calculations, boxplots, cluster analysis, and trend analysis using the Mann-Kendall statistical method. The seasonal variability of the average wind speeds of each group presented higher values for winter and spring and lower values in the summer and fall. The groups G1, G2, and G5 showed higher annual averages in the interannual variability of wind speeds. These observed peaks were attributed to the El Niño and La Niña events, which change the behavior of global wind circulation and influence wind speeds over the region. Trend analysis showed more significant negative values for the G3, G4, and G5 groups for all seasons of the year and in the annual average for the period under study.

## 1. Introduction

Information on wind speeds near the surface is used to assist in projects in various fields, such as in coastal erosion, pollutant dispersion, civil engineering, and the construction of wind farms for power generation. The growth of the world economy increases the demand for energy, and renewable energy sources, such as wind power, have proven to be a viable alternative that can be widely employed. 

Pereira et al. [1] reported that the production of wind energy in Brazil grew from 22 MW in 2003 to 602 MW in 2009. This can be attributed to incentive programs of the federal government, such as Incentive Program for Alternative Sources of Electric Power (*Programa de Incentivo às Fontes Alternativas de Energia Elética-*PROINFA). These authors found that according to the projections of the models of the last report of the Intergovernmental Panel on Climate Change (IPCC), scenarios A1 and B2 point to an increase in wind speeds exceeding 20% by 2100 in Northeastern Brazil (NEB), particularly in its northern and eastern parts.

To identify the wind potential of a location, it is necessary to have a time series with observations of wind speeds at a suitable height. In Brazil, the regions with the greatest potential are the coastlines of the South Atlantic, especially in the states of NEB, where the trade winds from the southeast (SE) in the Southern Hemisphere are strong. 

Recent studies have emphasized that climate change may affect wind speed trends. In China, a decrease in wind speeds has been observed between 1956 and 2004 [2], but these results may be the result of urbanization, which increases friction near the surface. In Australia, Troccoli et al. [3] considered two different periods (1975–2006 and 1989–2006) and observed a negative trend for wind speeds at 2 m and a positive one for those at 10 m from the surface. Other studies have also dealt with the climatological aspects of wind speeds over the continent at 10 m from the surface, employing statistical analyses, such as the clustering method, to identify homogeneous areas and long-term aspects of changes in wind speed [4–8].

Wind speed data obtained from meteorological stations or through numerical modeling has been used in different parts of the globe to identify the characteristics of seasonal and interannual variabilities [9–12]. As an explanation of the variability of wind speed results presented in this study, the study of Troccoli et al. [3] points to the following associated conditions: (i) the steep relief and aerodynamic roughness of the terrain; (ii) the presence of orography, causing thermal stability; (iii) the overlap of atmospheric circulation on different spatial scales (global, synoptic, mesoscale, and micro mesoescala), which influences the seasonal and interannual variabilities of the wind regime.

Some recent studies on wind speeds have revealed their importance for wind energy, which is also affected by climate change, as can be seen from the significant negative trends. Some examples are the studies by McVicar et al. [6] for the entire globe and by Cradden et al. [13] for the UK. 

It is known that NEB is a region with high wind potential. The studies that support this statement, however, have been realized with short data series, that is, less than 10 years. There are no studies on NEB with series of observational data exceeding 20 years that investigate climatological aspects and wind speed trends, neither are there studies on trends in recent years. Therefore, the objective of this paper is to investigate the climatological aspects and wind speed trends in NEB for a period of 26 years in 47 weather stations. We hope that the results presented here may contribute to the (scientific and political) discussion on the generation of renewable energy in Brazil.

## 2. Materials and Methods

### 2.1. Area of Study

The NEB region is situated at the easternmost end of South America, bathed in the north and east by the Atlantic Ocean. It is located in the NEB between the geographical coordinates 1°02′ to 18°20′ south and 34°20′ to 48°30′ west. It covers an area of 1.561.177.8 km^2^, which represents 18.3% of the Brazilian territory. Its population is 53.081.950 hab, divided over nine states: Alagoas (AL), Bahia (BA), Ceará (CE), Maranhão (MA), Paraíba (PB), Pernambuco (PE), Piauí (PI), Rio Grande do Norte (RN), and Sergipe (SE).

Rainfall in NEB is irregular, varying in both spatial and temporal distribution. The rainy season is concentrated between January and June, and the dry season stretches from July to December [14]. The climatological annual average is 1800 mm at the coast (coastal area) and below 400 mm in the central area (semiarid) [15]. According to the INMET, the average annual temperature in NEB varies from 20.7°C to 27.4°C. The maximum and minimum temperatures reach 33.8°C and 16.8°C, respectively. The annual average wind speed measured at 10 m varies from 0.5 to 5.5 m/s. 

### 2.2. Data

**Figure 1:**
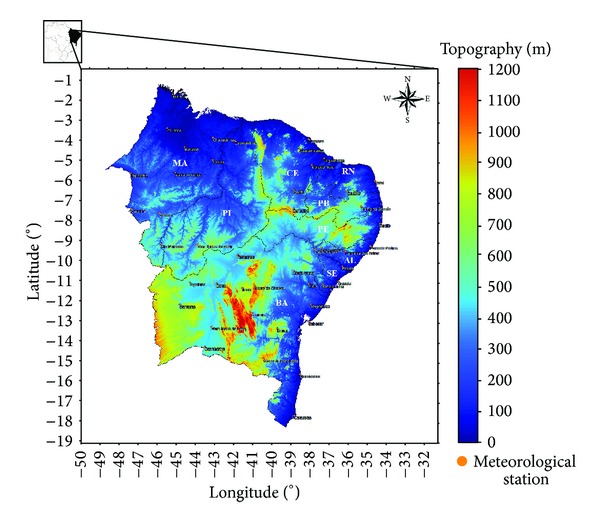
Spatial distribution of meteorological stations used in the study superimposed on the topography of NEB.

The wind speed data used were recorded by Fuess anemographs universal model AH-100 installed at 10 m above the surface and managed by the INMET. This equipment is intended to record the direction of the wind by a vane or arrow (pointing to the spot whence the wind) and wind speed throughout the day, with the three shells (http://www.inmet.gov.br/). The original set consisted of 92 anemographs; however, an inventory of each anemograph was made using the number of missing observations each year as an objective criterion. If the missing data values exceeded 15% of the total number of observations, the data of these anemographs was discarded, which reduced the set to 47 weather stations. The 1200 UTC was established as the time of observation, and the date was collected in the period from 1986 to 2011. The spatial distribution of the 47 stations used in this study is shown in Figure 1.

### 2.3. Filling in of Missing Data

We used the method of Multivariate Imputation by Chained Equations (MICE) to fill in the missing data. According to Van Buuren and Groothuis-Oudshoorn [16], the MICE technique can be used in various research areas, such as healthcare, politics, psychology, and sociology, or any other field of science that deals with missing data in their time series. The Predictive Mean Matching (PMM) was used to compensate for these missing data, using the data from the four geographically closest meteorological stations [17]. 

### 2.4. Cluster Analysis

Cluster analysis is an exploratory technique for multivariate data analysis that enables the classification of a set of observations into classes according to their similarities [5]. Cluster analysis was applied to the wind speed data for the period under study, using Ward's hierarchical classification method. For similarity or dissimilarity we used the Euclidean distance method. The Euclidean distance method is being increasingly used to identify homogeneous regions for wind speeds recorded at meteorological stations in various parts of the world [18–21]. 

**Figure 2:**
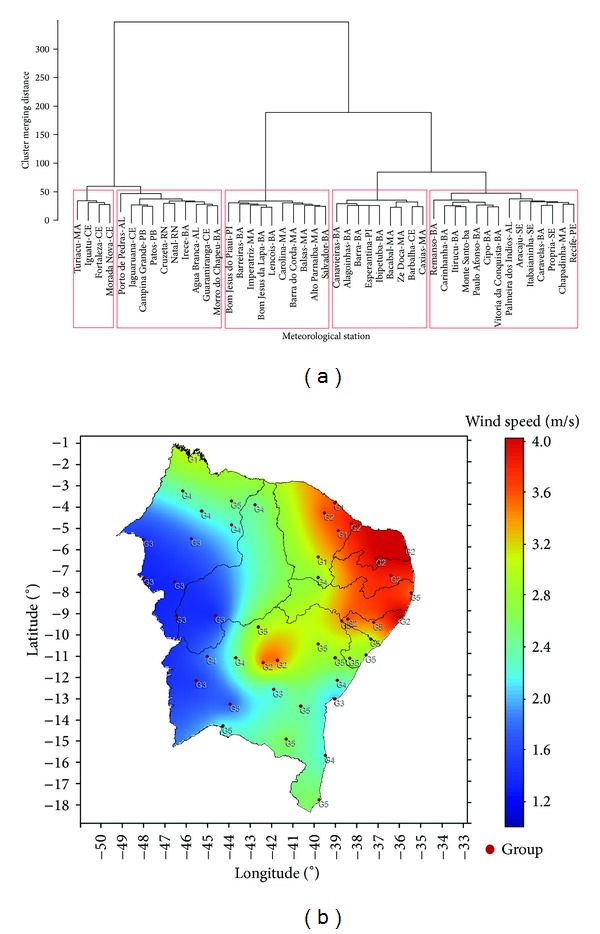
Formation of homogeneous groups for historical average wind speeds (a); (b) spatial distribution of the groups across NEB.

By applying cluster analysis, we were able to identify 5 homogeneous groups according to the monthly averages of the historical time series (1986–2011).  Figure 2(a) shows the dendrogram for the time series of monthly average wind speeds. It shows the connection of the locations with similar regimes. As can be seen in Figure 2(b), the geographical distance of the analyzed locations does not guarantee that the wind speed regimes are similar in data. 

In some cases, weather stations are clustered in the same group, even when they are in different regions of NEB. The weather stations of Salvador (BA) and Balsas (MA) are classified as G3, but they are separated by 1.023 km in a straight line. It is important to emphasize that the meteorological station in Salvador (BA) is located in the coastal area of NEB, in the State of Bahia. Its rainfall climatology is totally different from the weather station in Balsas (MA), which is located in the south of the state of Maranhão (Northern NEB).

### 2.5. Seasonal and Interannual Variabilities

There are several ways to study a one-dimensional data set. In this study, we applied the statistical boxplot technique described by Wilks [22] to establish the seasonal and interannual variabilities of wind speeds in the selected groups. This methodology includes information on estimated values, their location (mean or median), scale (interquartile range), and asymmetry (difference between quartile and median). Anomaly computation of intensity variations above or below the annual average wind speeds showed that the El Niño and La Niña events influence large-scale circulations, increasing or decreasing the intensity of winds over NEB.

### 2.6. Mann-Kendall Test

The nonparametric Mann-Kendall test has been suggested by the World Meteorological Organization (WMO) to assess the data trends in time series of environmental variables [23]. This test consists of comparing each value of the time series with the other values remaining in the sequential order. This test is based on the statistical term *S*, defined as follows:

(1) $$\begin{array}{c} S=\overset{n}{\underset{i=2}{\sum }}\overset{i-1}{\underset{j=1}{\sum }}\mathrm{sign}\left(x_{i}-x_{j}\right), \end{array}$$

where *x*_*j*_ are the values of the sequential data point; *n* is the length of the time series; sign⁡(*x*_*i*_ − *x*_*j*_) is −1 for (*x*_*i*_ − *x*_*j*_) < 0, 0 for (*x*_*i*_ − *x*_*j*_) = 0, and 1 for (*x*_*i*_ − *x*_*j*_) > 0. The mean *E*[*S*] and the variance *V*[*S*] are calculated according to the following equations:

(2) E[S]=0,VAR(S)=118[n(n−1)(2n+5)  −∑p=1qtp(tp−1)(2tp+5)],

where *t*_*p*_ is the number of connections to the *p*th value, and *q* is the number of connected groups. The values for *S* and VAR(*S*) are used to calculate the standardized *Z* test statistic as follows:

(3) $$\begin{array}{c} Z=\{\begin{array}{cc} \frac{S-1}{\sqrt{\text{VAR}\left(S\right)}} & \text{if}\;S>0\\ 0 & \text{if}\;S=0\\ \frac{S+1}{\sqrt{\text{VAR}\left(S\right)}} & \text{if}\;S<0. \end{array} \end{array}$$

The presence of a statistically significant trend is analyzed using the value of *Z*. A positive *Z* value indicates a positive trend, while a negative *Z* value points to a negative trend. To test the level of significance *α* of the trend increase or decrease, the *H*_0_ (null hypothesis) is rejected if the absolute value of *Z* is greater than *Z*_1−*α*/2_, where *Z*_1−*α*/2_ is obtained from the cumulative standard normal distribution tables [24]. In our case, the levels of the significance test *α* are 0.001, 0.01, 0.05, and 0.1.

## 3. Results

The mean, median, maximum, minimum, and standard deviations in the time series, separated by the groups selected in the cluster analysis, are shown in Table 1. The average wind speed is highest in the G1 (3.37 m/s), G2 (11.4 m/s), and G5 (2.83 m/s) groups and lowest in the G3 (1.35 m/s) and G4 (2.14 m/s) groups. The minimum speed varies from 0.30 m/s for group G4 to 1.29 m/s for G2, while the maximum ranges from 3.65 m/s for G3 to 7.71 m/s for G2. The variability (indicated by the standard deviation) is highest for G1 (0.97 m/s) and lowest for G3 (0.55 m/s).

### 3.1. Seasonal Variability

**Figure 3:**
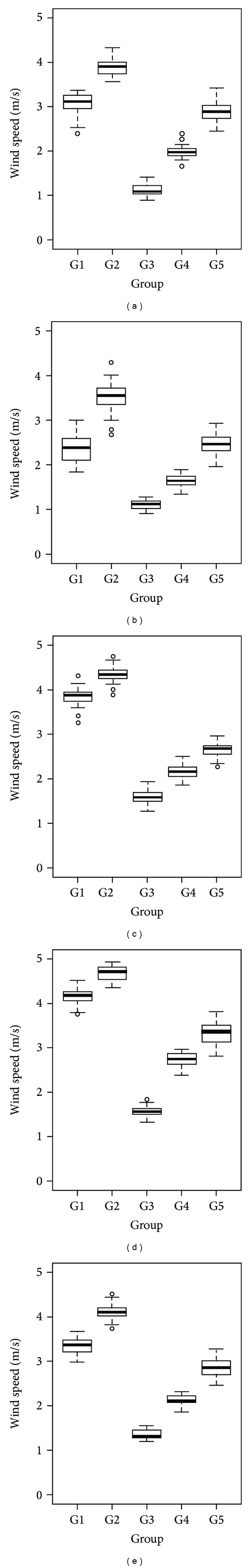
Boxplot showing the variability of the seasonal average wind speed of the groups: (a) summer, (b) autumn, (c) winter, (d) spring, and (e) the historical annual wind speed average.

In Figures 3(a)–3(e) the boxplots for the selected groups are traced. The boxplots for the historical average wind speeds for each season of the year suggest the presence of some apparently atypical values (outliers, represented by the symbol °), especially in winter (Figure 3(c)) for the G1 and G2 groups. In Figures 3(a)–3(d), we can observe that winter (June, July, and August-JJA) and spring (September, October, and November-SON) produce the highest wind speed values for the selected groups. G5 presents a higher median in spring with 3.3 m/s (Figure 3(d)), while in other seasons this value ranges between 2.50 and 2.86 m/s.

The highest median values were observed in the G1 and G2 groups during winter and spring, while the lowest values occurred in groups G3 and G4 during summer and autumn (March, April, and May-MAM). According to the boxplots values presented in Figures 3(a) and 3(b), groups G3 and G4 have less variability in wind speed for each seasonal transition. 

The variability of the average annual wind speeds of the groups for the period 1986–2011 is presented in the bloxplot of Figure 3(e). The largest variability around the median is presented by group G5, and the highest median values are observed in the groups G1, G2, and G5 (Figure 3(e)). The lowest variability is presented by groups G3 and G4, with a median equal to 1.27 and 2.6 m/s, respectively.

Group G2, which has meteorological stations located on the east coast and semiarid region of NEB, registers higher medians than the other groups (Figures 3(a)–3(e)). This is the region that is most influenced by trade winds, associated with the South Atlantic High and the sea breeze. G2 stands out in the comparison with other groups for all seasons, with winds averaging between 4.0 and 5.0 m/s. Another factor contributing to the high wind speed values in the G2 group is the topographic elevation of the semiarid region in NEB, which is defined by high plateaus. 

**Figure 4:**
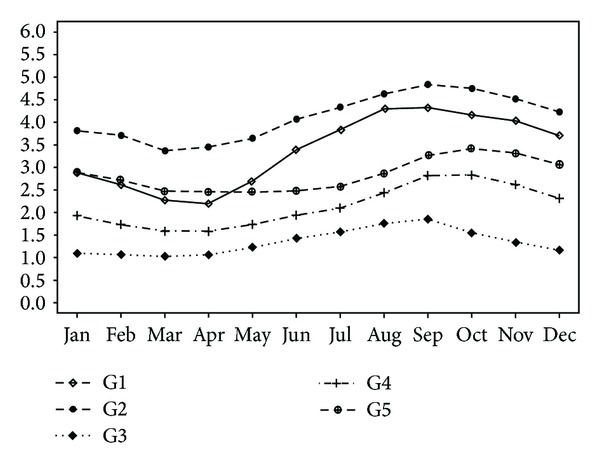
Average monthly wind speeds for the analyzed groups in the period from 1986 to 2011 in NEB.

The monthly cycle of the groups is shown in Figure 4. The minimum wind intensity values occur during the months corresponding with the rainy season in the NEB region, from February to May, and the maximum values occur in the months of August to October. 

### 3.2. Interannual Variability

**Figure 5:**
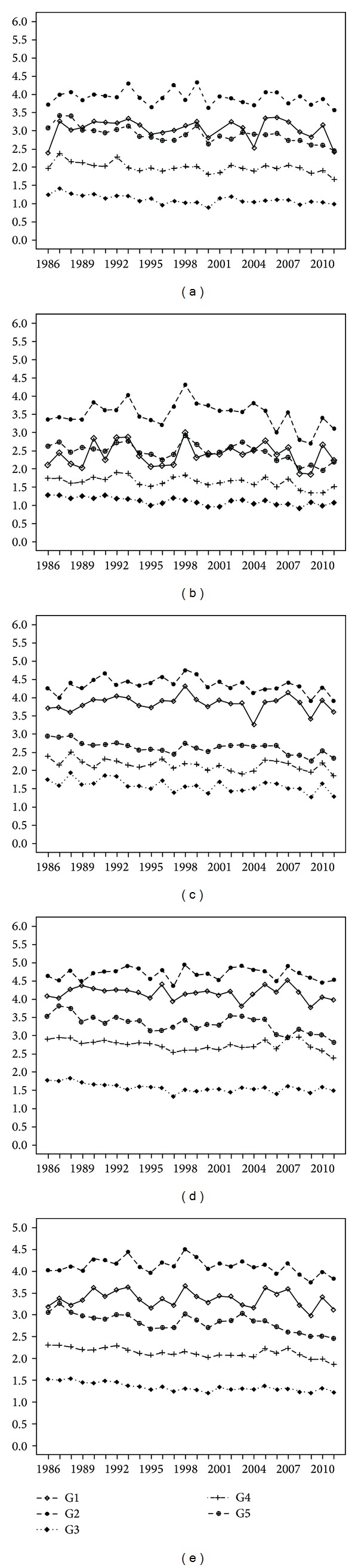
Quarterly ((a)–(d)) and annual (e) average wind speed graphs for the period 1986–2011.

The interannual analysis shows that the groups present higher values in the dry seasons (winter and spring) than in the rainy seasons (summer and fall), as can be seen in Figures 5(a)–5(e). The wind intensity of groups G1 and G2 is also influenced by the Intertropical Convergence Zone (ITCZ) localization. In August (winter) and September (spring), the land-ocean thermal gradients widen as the ITCZ migrates north. Consequently, the trade winds intensify through their joint action with the sea breeze. Conversely, in the rainy season (in particular, in the February-summer and March-autumn months), the movement of the ITCZ [9] to the south decreases wind speeds. Weather stations located in the south east coast of NEB belonging to the group G5 have lower wind speed values than those in groups G1 and G2 (Figures 5(c) and 5(d)) because of the weakening of the trade winds as a result of the localization of stations with respect to the equator, in combination with a moderate sea breeze (lower ocean-land thermal gradient). Winds in G5 intensify in spring. This is the dry season with its higher solar radiation and, consequently, a higher thermal gradient between ocean-land (sea breeze) associated with the trade winds (Figure 5(d)).

The low values observed for G3, in all seasons, with values below 2.0 m/s (Figures 5(a)–5(d)), is determined by the proximity of the weak pressure gradients associated with the equatorial depression [25], the high surface friction caused by its dense vegetation, and its relatively low topographical position. The same vegetation and topography factors apply to the weather stations of group G5.

Figure 5(e) presents the annual average wind speeds within the groups for the time series. We can observe that G1, G2, and G5 have the highest average wind speed values, while G3 and G4, with their own characteristics regarding circulation patterns and geomorphology, have the lowest annual average. In Figure 5(e), the variability of each year can be observed, with wind speeds staying above, below, or close to the historical average in a particular group. 

Table 2 presents the descriptive statistics of the interannual variability of the groups for the time series, taking into account the influence of El Niño and La Niña events on the change in wind intensity over NEB.

The 1987 El Niño, classified as moderate, is reflected in the maximum wind intensities of G1, G3, G4, and G5 (Figure 5(e)), which stayed above their historical averages (G1: 3.37 m/s, G3: 1.35 m/s, G4: 14.2 m/s, and G5: 2.83 m/s). These groups reach other maximums in the El Niño of 1993. In 1998, a strong El Niño event produced higher values than the historical averages for all groups (Table 2). 

In the La Niña events of 2000 and 2008, the wind speed intensity was always below the average, especially in 2000, with G3 presenting the largest anomaly: 0.15 m/s. In 2008, G5 was the biggest outlier with 0.25 m/s.

### 3.3. Trend Analysis

The trend analysis tests with the Mann-Kendall method are summarized in Table 3. We can observe that the average annual wind speeds for groups G3, G4, and G5 have a negative trend, with a significance level of *P* < 0.001, while G1 and G2 did not show any significance in their trend tests. 

In summer, the decrease in speeds was more pronounced in G3 and G5, with significance levels of *P* < 0.001. This trend was highest in group G5, with a *Z* value of −4.06, representing an impact of climate variability and on wind resources. In some locations of this group, wind intensity was greater than 3.0 m/s. 

Other important findings were the negative trend in winter and spring for G3 and G5. In G4, a negative trend can be observed with *Z* values of −2.64 and −2.51 and significance levels of *P* < 0.01 and *P* < 0.05 for winter and spring, respectively. This period is characterized by higher wind intensity, according to its climatology. The highest values in the Mann-Kendall trend test were found for summer and fall in all groups. For the rest of the analyses, significant levels of *P* < 0.001 and *P* < 0.01 were observed. 

## 4. Discussion and Final Remarks

In accordance with the study from Oliveira and Costa [26], the highest wind speeds were found in the period from August to November. When we look at the NEB region regarding its viability for wind energy projects, only groups G1 and G2 prove to be favorable locations, with historical averages above 3 m/s.

De Lucena et al. [27] used numerical weather models for future scenarios and presented results for the wind conditions in the northern coastline of NEB that prove favorable for investments in wind power. This could lead to an expansion in the use of renewable energy in this region. Three of the four weather stations in G1 are located in this area, and the wind speed values were considerable. NEB has a greater wind power potential in the second semester, especially the G1 and G2 groups. Pašičko et al. [28] argue in a detailed study that for the development of a climatologically viable wind farm project, wind speeds exceeding 3 m/s and in a constant direction are required. 

Based on the data analyzed in this study, we observed a seasonal variability in the groups of the NEB region, which can be seen in Figures 3 and 4. Lima and Filho [29, 30] have also demonstrated the existence of seasonality in wind speeds with data obtained from two anemometric towers, located in the central NEB region (semiarid) (Triumfo: 07050′17′′S, 38006′06′′W and São João do Cairi: 07022′54′′S, 36031′38′′W), with maximum values in the months of July to November and minimums in March and April. The seasonal wind speed variability of G1, G2, G3, G4, and G5 presented maximum and minimum values in these same months. In addition, Rehman [31] also confirmed seasonality in wind speed data collected at different points in Saudi Arabia, with the highest values occurring during the summer months (winter in the southern hemisphere) and the lowest during the winter months (summer in the southern hemisphere). 

Regarding the interannual variability in the groups, we observed that wind speeds increased during El Niño events and decreased during La Niña, which can be confirmed by the anomalies presented in Table 2. Vieira [32] observed an increase in wind speeds during the dry season along the coast of the state of Ceará of approximately 2 m/s for the El Niño year of 1983. In the strong La Niña event of 1999, he added to these results by observing that in the rainy season of the northern sector of NEB the average wind speed values decreased in relation to the climatological average.

Rehman [31] performed a statistical trend analysis with the Mann-Kendall method on the average annual data for the entire time series (1970–2006) from stations located in Saudi Arabia. The Al-Ahsa weather station presented a *Z* test value that indicates a decreasing annual average wind speed trend. Similar decreasing trends were observed in Al-Baha, Guriat, Sharourah, Taif, and Yanbo, with significant levels of *P* < 0.01, in addition to Gizan, Tabouk, Medinah, Nejran, and Qaisumah, which had a significance of *P* < 0.001. The G3, G4, and G5 groups present decreasing trends with *Z* test values and significance levels similar to those found by Rehman [31]. Pereira et al. [1] have shown a decreasing trend in their historical series of average annual wind speeds for weather stations in the NEB region (Caravels-BA; Parnaíba Sul region-PI; Maceió-AL). The results for G1 and G2 did not present significant annual trends in their results. Pereira et al. [1] state in their conclusion that the large number of nonsignificant results is a consequence of the few available meteorological stations with longer time series, which would enable more conclusive results.

Based on the results present, the conclusions main can be summarized in the following points.

- In the analysis of 47 meteorological stations of the NEB region, divided into five homogenous groups, the highest annual average wind speed (at 10 m from the surface) of 4.11 m/s was observed in G2 and the lowest was 1.35 m/s in G3.
- The highest median values for seasonal variability were observed in winter and spring, except for G5, which had its highest median value in spring with a value of 3.3 m/s. The variability of the average annual wind speeds in the boxplots showed a greater variability in group G5. The lowest variability was presented by G3 and G4. The groups with the highest median values were G1, G2, and G5. We also found that the locations of G2 with an elevated topography, specifically central NEB (semiarid), favor an increase in wind intensity.
- The G1, G2, and G5 groups presented the highest annual averages for interannual variability. The lowest were observed in G3 and G4. We found that in the years 1987 (G1, G3, G4, and G5), 1993 (G1–G5), 1998 (G1, G2, G4, and G5), and 2005 (G1–G5), the average wind speeds were above the historical average. The intensification of circulation in the NEB region for these years is caused by El Niño events. During the La Niña events of the years 1988 (G1 and G2), 2000 (G1–G5), and 2008 (G1–G5), the annual average speeds remained below their historical averages.
- The analysis of wind speed trends enables us to draw the following conclusions: (i) the groups G3, G4, and G5 showed a negative trend in annual average speeds with a high significance (*P* < 0.001); (ii) no significant trend was identified for groups G1 and G2; (iii) during the summer, a more pronounced decrease in wind speeds was observed in G3 and G5, with a significance level of *P* < 0.001 and *Z* test values of −3.61 and −4.06; (iv) in winter and spring, group G4 presented the strongest negative trend, with *Z* values of −2.64 and −2.52 and significant levels of *P* < 0.01 and *P* < 0.05, respectively; (vi) the highest *Z* values were found in the summer and fall for all groups.
- The study indicates that the regions G1 and G2 have the greatest potential for expanding the use of wind power, since these are the areas with high wind speeds and no significant trends;
- It should be noted that these results were obtained from conventional meteorological stations at a specific time (12 *Z*). This analysis should therefore be extended, and, at the same time, the results portrayed here should be interpreted with caution. Improvements can be made by including a greater number of wind speed data measured by conventional (at four times: 00, 06, 12, 18 *Z*) and automatic (every hour) meteorological stations in NEB. These stations should also have a lower percentage of missing data. We believe, however, that the results presented here are of great value for the planning of future investments in wind power in NEB.

## Figures and Tables

**Table 1:** Descriptive statistical analysis for each group of the time series.

Group	Period	Minimum (m/s)	Maximum (m/s)	Median (m/s)	Mean (m/s)	Standard deviation (m/s)
G1	1986–2011	0.60	5.89	3.47	3.37	0.97
G2	1986–2011	1.29	7.71	4.07	4.11	0.84
G3	1986–2011	0.10	3.65	1.27	1.35	0.55
G4	1986–2011	0.30	4.50	2.06	2.14	0.64
G5	1986–2011	1.17	6.63	2.78	2.83	0.72

**Table 2:** Years with El Niño and La Niña events, which are responsible for changes in wind intensity over the studied region. Years with El Niño and La Niña activities were established based on the data from the Center for Weather Forecasting and Climate Studies-CPTEC/INPE (http://enos.cptec.inpe.br/).

Meteorological phenomenon	Year	Observed mean (m/s)					Anomaly (m/s)				
		G1	G2	G3	G4	G5	G1	G2	G3	G4	G5
El Niño	1987	3.38	4.02	1.51	2.31	3.27	0.01	−0.09	0.16	0.17	0.44
La Niña	1988	3.22	4.10	1.54	2.27	3.06	−0.15	−0.01	0.19	0.13	0.23
El Niño	1993	3.64	4.44	1.37	2.19	3.00	0.27	0.38	0.02	0.05	0.17
El Niño	1998	3.67	4.51	1.31	2.16	3.02	0.30	0.40	−0.04	0.02	0.19
La Niña	2000	3.27	4.06	1.20	2.02	2.71	−0.10	−0.05	−0.15	−0.12	−0.12
El Niño	2005	3.62	4.15	1.37	2.22	2.86	0.25	0.04	0.02	0.08	0.03
La Niña	2008	3.22	3.91	1.23	2.08	2.58	−0.15	−0.20	−0.12	−0.06	−0.25

**Table 3:** Results of the Mann-Kendall tests of the groups for seasonal and interannual variabilities.

Group	Period	Average (m/s)	Mann-Kendall test (*Z*)	Signific. (*P*)
G1	Annual	3.37	−0.44	—
	DJF (summer)	3.05	−0.62	—
	MAM (autumn)	2.39	0.26	—
	JJA (winter)	3.84	0.26	—
	SON (spring)	4.17	−1.28	—

G2	Annual	4.11	−1.59	—
	DJF (summer)	3.91	−1.50	—
	MAM (autumn)	3.49	−1.28	—
	JJA (winter)	4.34	−1.54	—
	SON (spring)	4.70	−0.22	—

G3	Annual	1.35	−4.14	<0.001
	DJF (summer)	1.11	−3.61	<0.001
	MAM (autumn)	1.12	−4.01	<0.001
	JJA (winter)	1.59	−2.73	<0.01
	SON (spring)	1.58	−3.44	<0.001

G4	Anual	2.14	−4.10	<0.001
	DJF (summer)	1.99	−2.64	<0.01
	MAM (autumn)	1.64	−2.82	<0.01
	JJA (winter)	2.16	−2.64	<0.01
	SON (spring)	2.75	−2.51	<0.05

G5	Anual	2.83	−4.14	<0.001
	DJF (summer)	2.90	−4.06	<0.001
	MAM (autumn)	2.46	−3.09	<0.01
	JJA (winter)	2.64	−3.92	<0.001
	SON (spring)	3.33	−3.26	<0.01
